# Phlebosclerotic colitis: an analysis of clinical and CT findings in 29 patients with long-term follow-up

**DOI:** 10.1186/s13244-022-01159-x

**Published:** 2022-01-29

**Authors:** Sheung-Fat Ko, Hong-Hwa Chen, Chung-Cheng Huang, Li-Han Lin, Shu-Hang Ng, Yi-Wei Lee

**Affiliations:** 1 Department of Radiology, Kaohsiung Chang Gung Memorial Hospital and Chang Gung University College of Medicine, 123 Ta-Pei Road, Niao-Sung District, Kaohsiung, 833 Taiwan; 2 Division of Colorectal Surgery, Department of Surgery, Kaohsiung Chang Gung Memorial Hospital and Chang Gung University College of Medicine, Kaohsiung, Taiwan

**Keywords:** Mesenteric veins (calcifications), Mesentery (phlebosclerosis), Colitis (phlebosclerosis), Colitis (ischemia), Tomography (X-ray computed)

## Abstract

**Background:**

Phlebosclerotic colitis (PC) is a rare form of nonthrombotic colonic ischemia. This retrospective study analyzed the clinical findings and temporal CT changes in 29 PC patients with long-term follow-up.

**Methods:**

Twenty-nine patients with characteristic CT features of PC collected between 1997 and 2020 were stratified into the acute abdomen group (AA-group) (*n* = 10), chronic-progressive group (CP-group) (*n* = 14) and chronic-stable group (CS-group) (*n* = 5). Clinical and CT changes during follow-up, comorbidities and final outcomes were compared.

**Results:**

The AA-group exhibited a significantly thicker colonic wall and more involved segments and pericolic inflammation than the CP-group and CS-group on initial CT (*p* =  < 0.001–0.031). Seven patients in the AA-group who underwent right hemicolectomy had no recurrence during follow-up (mean ± SD, 7.1 ± 3.3 years), and the remaining three patients with renal or hepatic comorbidities who underwent conservative treatment died within 14 days. The CP-group showed significantly higher frequencies of chronic renal failure, urinary tract malignancies and liver cirrhosis than the AA-group (*p* = 0.005–0.008). In addition, CT follow-up (7.9 ± 4.3 years) showed significant increases in mesenteric venous calcifications, colonic wall thickening and involved colonic segments (*p* = 0.001–0.008) but conservative treatments were effective. The CS-group remained unchanged for years (8.2 ± 3.9 years).

**Conclusions:**

Early surgery offered excellent prognosis in PC-related acute abdomen denoted by marked right colonic wall thickening and pericolic inflammation on CT. Conservative treatments with a wait-and-watch strategy were appropriate for CP-PC and CS-PC, albeit CP-PC harbored significant increases in calcifications, colonic wall thickening and affected segments in long-term CT follow-up.

## Key points

- Clinical manifestations of phlebosclerotic colitis (PC) are nonspecific, and CT permits definitive diagnosis by demonstration of characteristic curvilinear mesenteric venous calcifications.
- Early surgery offered excellent prognosis in PC-related acute abdomen denoted by marked right colonic wall thickening and pericolic inflammation on CT.
- Chronic-progressive PC harbored frequent comorbidities and significant increases in calcifications, colonic wall thickening and affected colonic segments seen in long-term CT follow-up.
- Conservative treatments with a wait-and-watch strategy and CT follow-up were appropriate for chronic-progressive and chronic-stable PC.

## Background

Phlebosclerotic colitis (PC) is an unusual form of colonic ischemia histopathologically characterized by noninflammatory extensive fibrotic sclerosis of the thickened venous wall, prominent calcifications within stenotic venous lumen and fibrosis of the lamina propria and/or submucosa of the right colon [1–13]. The disease was first reported as chronic ischemic colitis by Koyama et al. in 1991 [1], designated PC by Yao et al. in 2000 and mesenteric phlebosclerosis by Iwashita et al. in 2003 [2, 3]. Until 2020, approximately 200 PC cases were documented in the literature with markedly high predominance in patients of Asian ancestry and a prevalence of 0.01 cases per 100,000 people in Japan [4–14]. Various tools, including plain radiography, barium enema, angiography, colonoscopy and CT, have been utilized to assess PC [2, 5–11]. CT is particularly useful because it offers a demonstration of distinctive right colonic curvilinear or threadlike mesenteric venous calcifications, assessment of potentially fatal complications such as peritonitis, bowel necrosis or perforation and noninvasive follow-up [5–8]. Recent CT studies on PC have focused on prediction of surgery and the relationship between the degree of calcification and colonic wall thickness or disease severity [9–11]. However, a great variety of clinical manifestations of PC ranging from severe abdominal pain to asymptomatic status have been recognized [8–13], reflecting that PC may exhibit diverse disease courses necessitating different treatment strategies. To our knowledge, long-term changes of clinical and CT features of PC have not been reported. This retrospective study aimed at analyzing the clinical findings and temporal CT changes in 29 PC patients with long-term follow-up to elucidate the variable natural history of this extraordinary condition.

## Methods

### Patients

The computer tomography database in our department and the picture archiving and communication system in our institution (a tertiary medical center) were searched from January 1997 to July 2020. Records with keywords including mesenteric vascular or venous calcifications, PC, mesenteric phlebosclerosis, ischemic colitis or colonic ischemia in the CT reports were collected, and the images were reviewed. Firstly, patients who were radiologically suspected of having PC were enrolled based on the following inclusion criteria: (1) the patient had unenhanced or enhanced abdominal CT with demonstration of characteristic right colonic curvilinear venous calcifications (> 100 Hounsfield units) along the tributaries of the mesenteric vein [2–12]; (2) for patients managed with conservative treatments, at least one CT follow-up ≥ 2 years after initial diagnosis was available. The exclusion criteria were PC patients underwent medical treatment without CT follow-up ≥ 2 years after initial diagnosis, or poor CT image quality. The medical records of the enrolled patients were reviewed. The age, sex, clinical manifestations, comorbidities, laboratory data, imaging features, surgical records and final outcomes were recorded. Secondly, the enrolled patients were then stratified into 3 groups according to clinical manifestations. The acute abdomen group (AA-group) included those who presented with acute severe abdominal pain, nausea, rebound tenderness and guarding. The chronic-progressive group (CP-group) included those who had initial abdominal pain or discomfort that was relieved by conservative treatments, while slow progression of clinical symptoms and abnormal CT findings were found during follow-up. The chronic-stable group (CS-group) included those who had mild abdominal symptoms or asymptomatic with stable clinical status and CT findings during follow-up. Our institutional review board approved this retrospective case review study (approval number: 202101197B0). The need for written informed consent from the patients was waived by the board due to the retrospective and anonymous nature of the analysis.

### Abdominal CT acquisitions

Abdominal CT examinations were performed with single-detector scanners (General Electric 9800 or ProSpeed scanners; GE Healthcare) and multi-detector scanners (Lightspeed, GE Healthcare; Aquilion 64, Toshiba Medical Systems; Somatom Volume Zoom, Somatom Definition AS, Somatom Definition Flash, Siemens). Each examination included unenhanced and/or contrast material-enhanced acquisitions with a scanning range from the liver dome to the pelvic floor. Iodinated contrast medium (350 mgI/mL) was administered with a bolus injection (rate, 2–3 mL/s; volume, 70–100 mL) by using a power injector. Scanning parameters included field of view 300–350 mm, matrix 512 × 512, 100–130 kVp, 150–280 mAs, rotation time 0.5 s and spiral pitch factor 0.6–1.2 for multi-detector CT, axial and coronal reconstructions at 5–10 mm section thickness. For those patients who underwent multi-detector CT angiography (CTA), maximum intensity projection (MIP) display and multiplanar reconstruction were also performed.

### Assessment of radiological findings

All abdominal radiographs, barium enemas, abdominal angiograms and CT scans were reviewed by two experienced radiologists (each with more than 20 years of experience in reading abdominal CT and emergency imaging) who were familiar with CT features of PC but were blinded to the clinical information and disease progress of the patients. Any discrepancies were resolved by consensus. The following CT features were specifically assessed: (1) degree of mesenteric venous calcifications, (2) maximum thickness of affected colon, (3) number of colonic segments involved and (4) presence of pericolic inflammation (denoted by prominent pericolic edema and fat stranding). The degree of calcifications in PC was assessed according to a 4-grade scoring method proposed by Yen et al. [9]. Each calcified mesenteric venous branch was divided into four segments: the straight vein, the marginal vein and the proximal and distal half of the main branch of the mesenteric vein. Involvement of each of these four segments was scored as 1, 2, 3 and 4, respectively. The severity of calcifications of PC on CT was defined as the summation of total scores of each calcified segment of all calcified mesenteric venous branches. The maximum thickness of the affected colon was defined as the averaged thickness of the thickest parts of each involved colonic segment. The colon was divided into 7 segments: cecum to proximal third ascending colon, distal two-third ascending colon, hepatic flexure, transverse colon, splenic flexure, descending colon and sigmoid colon.

### Data and statistical analysis

Clinical data of all patients, comprising age, sex, frequencies of comorbidities (including diabetes mellitus, chronic renal failure [patients with estimated glomerular filtration rate < 30 mL/min/1.73m^2^], urotract malignancies, liver cirrhosis or hepatocellular carcinoma), usage of herbal medication, leukocyte counts and C-reactive protein level, follow-up durations, initial and follow-up CT findings, PC calcification score, maximum colonic wall thickness, number of affected colonic segments and frequency of the presence of pericolic inflammation) and outcome, were recorded.

Comparisons and statistical analyses between the AA-, CP- and CS-groups were performed with IBM SPSS Statistics (version 26.0). A nonparametric approach was used in the analysis due to the limited sample sizes. The Kruskal–Wallis test (ordinal) or chi-square test (categorical) was used to determine the differences. For items with a *p* value ≤ 0.05, further analysis using the Mann–Whitney *U* test with Bonferroni correction or the chi-square test was employed for intergroup comparisons. For each of these intergroup comparisons using the chi-square test with a total of 3 comparisons for each variable of interest, the significance level was set at approximately 0.02 (0.05/3 = 0.017) because multiple comparisons tend to increase type I error. The Mann–Whitney *U* test or chi-square test was employed for intergroup comparisons of the findings in initial CT of AA-group vs final CT of CP-group and final CT of CP-group vs CS-group. The Wilcoxon signed rank test was employed for comparisons of initial and final CT findings in the CP- and CS-groups. A *p* value ≤ 0.05 was considered to indicate a statistically significant difference.

## Results

### Patients

**Fig. 1:**
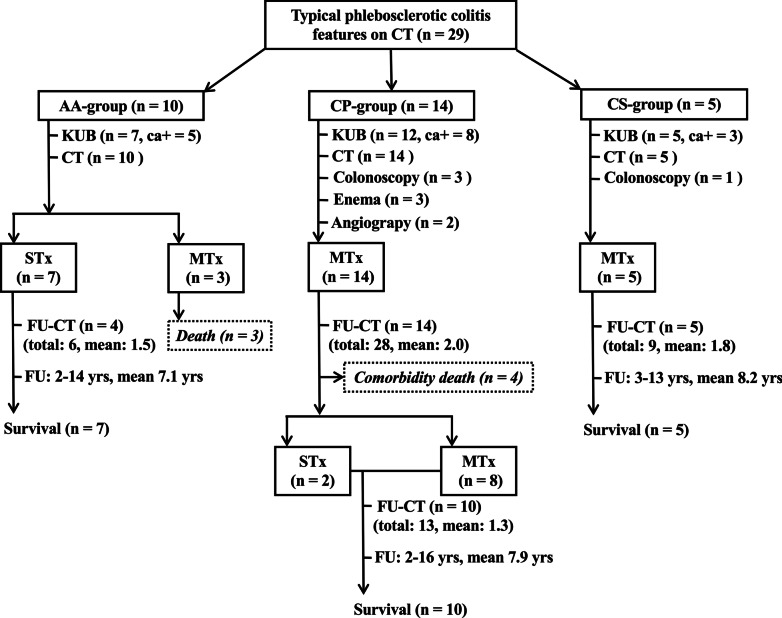
Selected treatment, radiological examinations, colonoscopy and outcomes in three groups of patients with PC. ca (+), curvilinear calcifications positive; STx, surgical treatment; MTx, medical treatment; FU, follow-up; total, total number of CT examinations

Out of 588 patients initially collected with positive keywords in the CT reports, 29 patients fulfilled the inclusion criteria were enrolled. Three other medically treated PC patients, one without CT follow-up, one with CT follow-up 11 months after initial diagnosis and one with poor CT quality, were excluded. Among 29 enrolled PC patients (7 men, 22 women; age range 31–74 years, mean 60.1 years), a great variety of clinical presentations were noted. The AA-group (*n* = 10) presented with severe abdominal pain in 10, tenderness and muscle guarding in 8, nausea in 6 and vomiting in 2 patients, but prior intermittent mild abdominal pain was found in 6 patients. The CP-group (*n* = 14) presented with dull abdominal pain in 12, diarrhea in 5, abdominal distension in 2 and tarry stool in 2 patients. The CS-group (*n* = 5) presented with dull abdominal pain in 2 patients and one of them had intermittent diarrhea, while the other 3 patients were asymptomatic. The treatment methods, CT, enema, angiography, colonoscopy and clinical outcomes of 29 patients with PC are summarized in Fig. 1. Curvilinear calcifications in the right abdomen could be identified in 16 out of 24 available initial abdominal radiographs. Three patients displayed limited distension, effacement of the haustral folds and focal thumb printing of the right colon in barium enemas. Two out of 4 patients who underwent colonoscopy showed bluish-purple-colored mucosa with focal ulcerations in the right colon, while two other patients were initially diagnosed with ulcerative colitis. Superior mesenteric arteriograms in 2 patients demonstrated mildly tortuous marginal arteries and vasa recti of the right and middle colic arteries and nonopacification of the middle colic and marginal venous lumens in the portal phase with some faintly opacified collateral veins. The AA-group showed trends of higher leukocyte counts (12,600 ± 3900 cells/µL, mean ± SD vs 10,800 ± 4800 cells/µL or 7500 ± 2300 cells/µL) and C-reactive protein levels (13.5 ± 8.2 mg/dL, mean ± SD vs 10.4 ± 6.8 mg/dL or 4.6 ± 0.9 mg/dL) than the CP-group and CS-group, respectively (*p* = 0.051–0.053).

### Comparison of comorbidities and CT findings

**Fig. 2:**
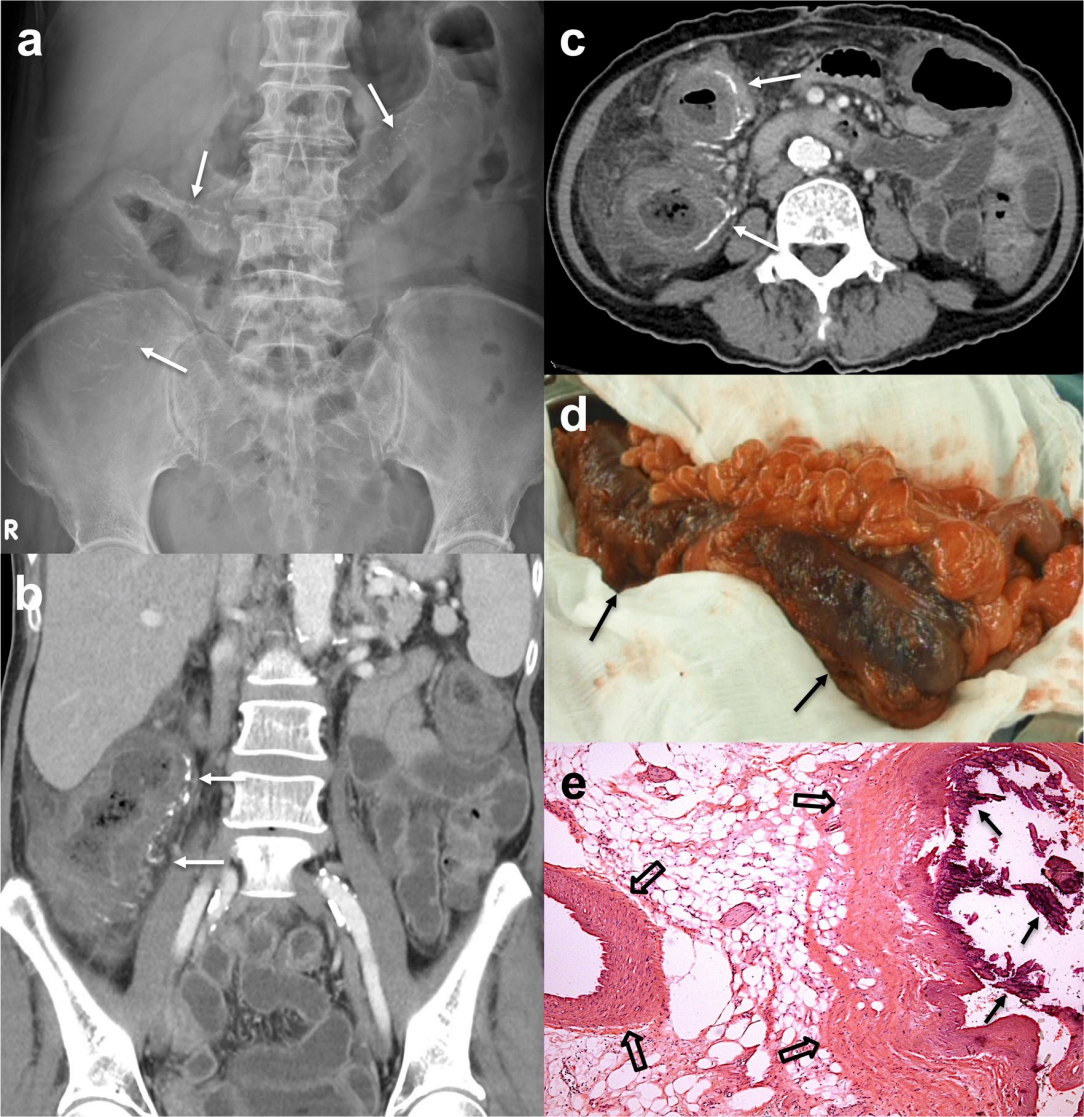
A 72-year-old woman was admitted due to acute abdomen with severe abdominal pain for 2 days with tenderness and muscle guarding on palpation. **a** Plain radiograph shows curvilinear calcifications (arrows) alongside the right colon. **b**, **c** Axial and coronal CT show curvilinear calcifications of the straight and marginal veins alongside the ascending colon with thickened walls, prominent pericolic edema and inflammatory stranding. **d** Surgery revealed a swollen right colon with dark-purple discoloration. **e** Photomicrograph (H and E, × 40) shows mesenteric veins (open arrows) with marked fibrosclerotic wall thickening and intraluminal calcifications (black arrows), confirming PC

**Fig. 3:**
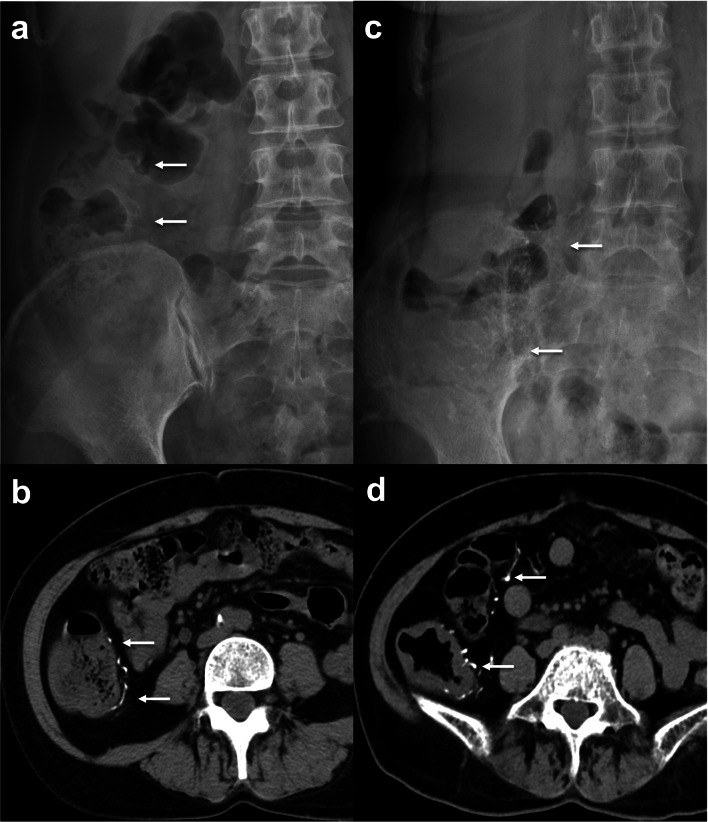
A 51-year-old woman with bladder cancer incidentally found mesenteric venous calcifications. **a**, **b** Plain radiograph and abdominal CT show curvilinear calcifications (arrows) of the straight veins of the cecum and proximal ascending colon. **c**, **d** Intermittent diarrhea noted for 7 years and follow-up radiograph and CT show progression of mesenteric venous calcifications (arrows) and cecal wall thickening without pericolic inflammation

Table 1 summarizes the age, sex, comorbidities, follow-up durations and initial CT findings in the 3 groups of patients with PC. There were no significant differences in age or sex, but female preponderance (70–80%) was noted in all three groups. Only 6 patients had frequent usage of herbal medications. The AA-group had significantly lower frequencies of chronic renal failure, urotract malignancies and liver cirrhosis than the CP-group (*p* = 0.005–0.008). Seven out of 14 patients in the CP-group had concurrent chronic renal failure and liver cirrhosis, and among them, urotract malignancies were found in 5 patients. The AA-group exhibited a significantly thicker colonic wall, more involved colonic segments and higher frequencies of pericolic inflammation than the CP-group and CS-group on initial CT (*p* =  < 0.001–0.031) (Fig. 2). There were no significant differences of initial CT findings between CP- and CS-groups. Table 2 summarizes the initial and final follow-up CT findings. Twenty-three patients (4 in the AA-group, all patients in the CP- and CS-groups) with a total of 56 follow-up CT scans (ranging from 1 to 5 times in 2–16 years) were available for comparison. Compared with the initial CT, the CP-group showed significant increases in calcification scores, colonic wall thickness and involved colonic segments on follow-up CT (*p* = 0.001–0.008) (Fig. 3).

**Table 1:** Comparisons of the clinical and initial CT findings between the AA-group, CP-group and CS-group with phlebosclerotic colitis

	AA-group (*n* = 10)	CP-group (*n* = 14)	CS-group (*n* = 5)	P1	P2	P3	P4
**Clinical findings**							
Age (years) (mean ± SD)	58.1 ± 14.5	62.0 ± 7.3	58.0 ± 9.8	NS			
Gender, female, *n* (%)	7 (70%)	11 (76%)	4 (80%)	NS			
Diabetes (+), *n* (%)	1 (10%)	6 (43%)	0 (0%)	NS			
Chronic renal failure (+), *n* (%)	2 (20%)	10 (71%)	1 (20%)	0.011^‡^	0.005^‡^	NS	NS
Urotract malignancies (+), *n* (%)	2 (20%)	9 (64%)	1 (20%)	0.017^‡^	0.008^‡^	NS	NS
Liver cirrhosis (+), *n* (%)	2 (20%)	9 (64%)	1 (20%)	0.017^‡^	0.008^‡^	NS	NS
Hepatocellular carcinoma (+), *n* (%)	2 (20%)	2 (14%)	0 (0%)	NS			
Herbal medicine > 5 years (+), *n* (%)	2 (20%)	3 (21%)	1 (20%)	NS			
Follow-up duration (years) (mean ± SD)	7.1 ± 3.3	7.9 ± 4.3	8.2 ± 3.9	NS			
**Initial CT findings**							
PC calcification scores (mean ± SD)	23.5 ± 8.4	14.7 ± 10.9	11.8 ± 5.7	0.046^*^	NS	NS	NS
MWT(mm) (mean ± SD)	10.5 ± 3.4	2.3 ± 1.2	1.6 ± 0.5	< 0.001^*^	< 0.001^§^	0.001^§^	NS
PC segments (mean ± SD)	4.0 ± 0.8	2.4 ± 1.7	2.2 ± 1.1	0.047^*^	0.031^§^	0.028^§^	NS
Pericolic inflammation (+), *n* (%)	10 (100%)	0 (0%)	0 (0%)	< 0.001^‡^	< 0.001^‡^	< 0.001^‡^	NS

PC, phlebosclerotic colitis; MWT, maximum colonic wall thickness; (+), positive; NS, not significant

P1: *Kruskal–Wallis test (numeric) or ^‡^Chi-square test (nominal) for 3 groups. If P1 < 0.05, further analysis using the ^§^Mann–Whitney *U* test with Bonferroni correction or ^‡^Chi-square test

P2: AA- vs CP-groups, P3: AA-vs CS-groups, P4: CP- vs CS-groups

**Table 2:** Comparisons of follow-up CT findings between the AA-group, CP-group and CS-group with phlebosclerotic colitis

	AA-group (*n* = 10)	CP-group (*n* = 14)	CS-group (*n* = 5)	P2	P3
**PC calcification scores (mean ± SD)**					
Initial	23.5 ± 8.4	14.7 ± 0.9	11.8 ± 5.7		
Final	NA	25.6 ± 10.1	12.1 ± 5.8	NS	0.010^*^
*P1*		*0.001*^§^	*NS*		
**MWT(mm) (mean ± SD**)					
Initial	10.5 ± 3.4	2.3 ± 1.2	1.6 ± 0.5		
Final	NA	5.3 ± 1.4	1.7 ± 0.6	< 0.001*	< 0.001*
*P1*		*0.002*^§^	*NS*		
**PC segments (mean ± SD)**					
Initial	4.0 ± 0.8	2.4 ± 1.7	2.2 ± 1.1		
Final	NA	3.9 ± 1.9	2.2 ± 1.1	NS	NS
*P1*		*0.008*^§^	*NS*		
**Pericolic inflammation positive, *n* (%)**					
Initial	10 (100%)	0 (0%)	0 (0%)		
Final	NA	2 (14%)	0 (0%)	< 0.001^‡^	NS
*P1*		*NS*	*NS*		

PC, phlebosclerotic colitis; MWT, maximum colonic wall thickness; initial, initial CT; final, final follow-up CT, NA, not applicable for post-operative CT follow-up of AA-group; NS, not significant

*Mann–Whitney *U* test, ^‡^Chi-square test, ^§^Wilcoxon signed rank test

P1: initial CP- vs final CP-group, initial CS- vs final CS-group

P2: initial AA- vs final CP-group

P3: final CP- vs final CS-group

**Fig. 4:**
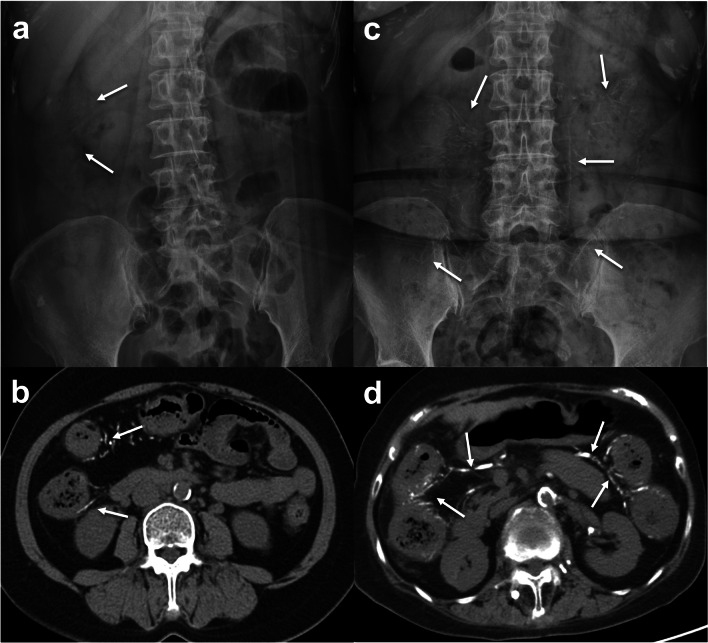
A 58-year-old woman with hepatitis B and chronic renal failure initially presented with mild right abdominal discomfort. **a**, **b** Plain radiograph and abdominal CT show curvilinear calcifications (arrows) alongside the distal ascending colon. **c**, **d** Mild abdominal pain and intermittent tarry stool noted for 11 years and follow-up radiograph and CT show progression of PC with calcification of superior and inferior mesenteric venous branches (arrows) and colonic wall thickening extending to distal descending colon

**Fig. 5:**
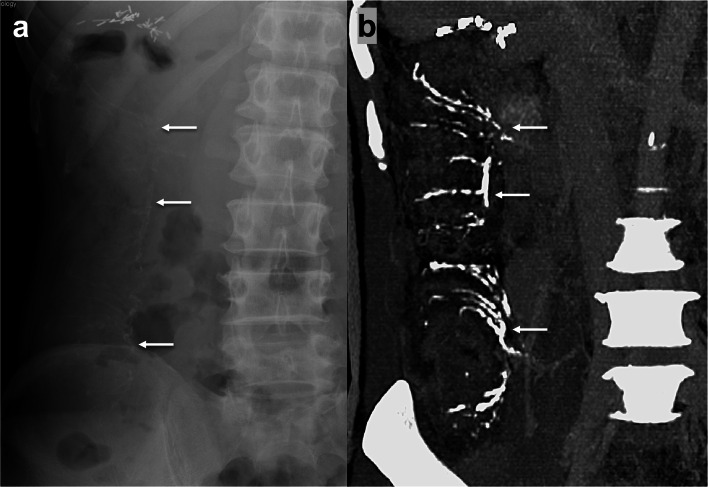
**a** Curvilinear calcifications alongside the right colon seen on abdominal radiograph. **b** Corresponding maximum intensity projection display of CT angiography shows comprehensive demonstration of the calcified straight and marginal veins of the ascending colon

**Fig. 6:**
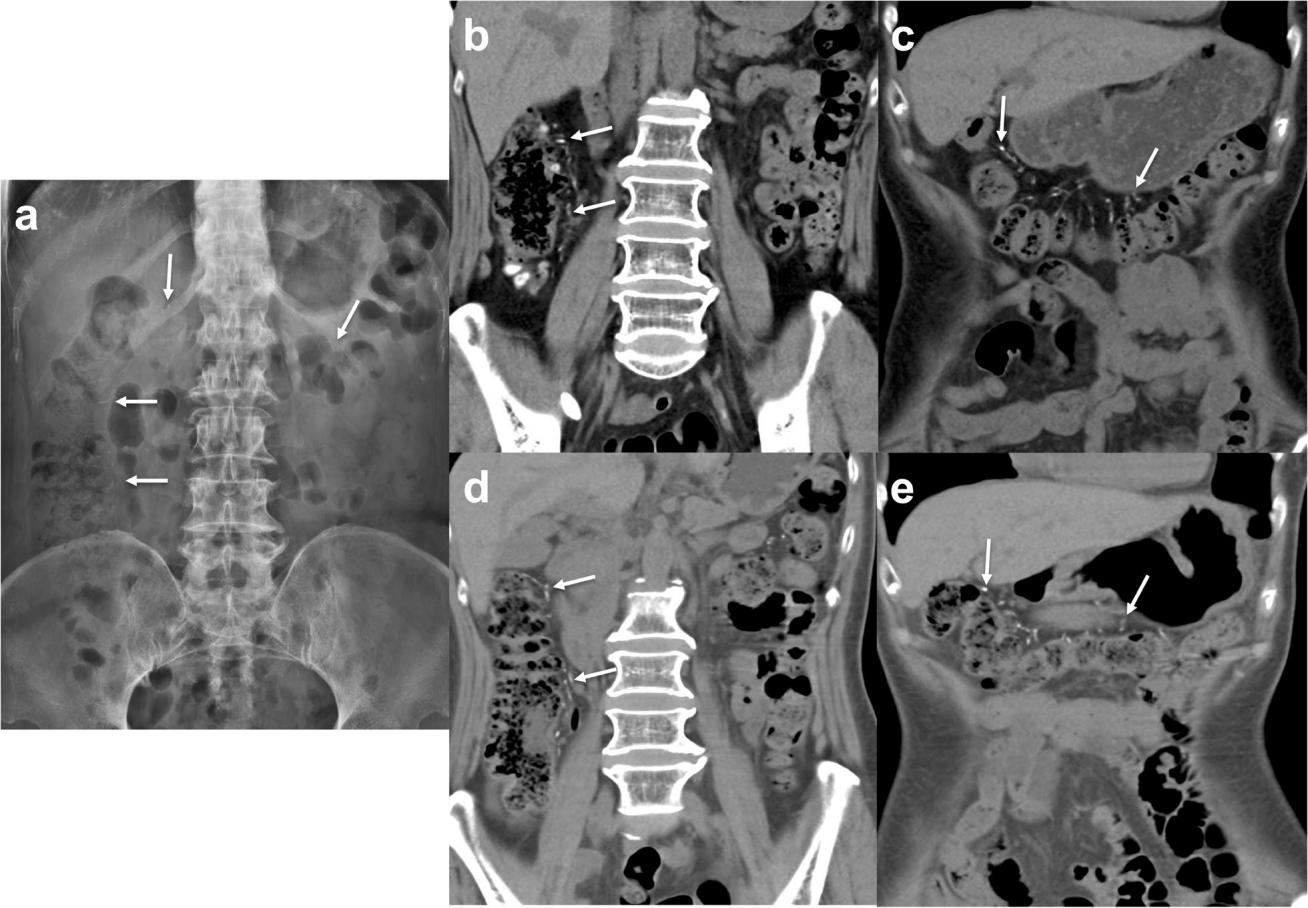
A 52-year-old asymptomatic woman had a past history of hysterectomy for early endometrial cancer. **a** Curvilinear calcifications alongside the right colon were incidentally observed on abdominal radiograph. **b**, **c** CT confirming calcified straight and marginal veins of the right colon without colitis. **d**, **e** Five-year follow-up CT shows stable calcified mesenteric veins with no colonic wall thickening

Progression of PC to the descending colon in 3 patients and sigmoid colon in 1 patient with calcification of the superior and inferior mesenteric veins were found in the CP-group (Fig. 4). CTA with MIP display were also available in 3 CP-patients with explicit delineation of venous calcifications (Fig. 5) making the assessment of calcification score easier but the total score remained the same as those obtained from conventional CT images. Follow-up CT of the CP-group also showed significantly higher calcification scores and thicker colon walls than those of the CS-group. However, colonic wall thickening and frequencies of pericolic inflammation in follow-up CT of the CP-group were still significantly less than those seen in initial CT of the AA-group. The CS-group showed no significant changes between the initial and follow-up CT (Fig. 6).

### Follow-up and outcomes

There were no significant differences of follow-up durations between the AA-, CP- and CS-groups (Table 1). Seven out of 10 patients in the AA-group who underwent right hemicolectomy within 10 days recovered well. Histopathological examinations confirmed the diagnosis of PC with extensive fibrotic sclerosis of the thickened venous wall and prominent calcifications within stenotic lumen (Fig. 2e). CT follow-up in 4 patients showed no new calcifications or wall thickening in the residual colon and none with PC-related symptoms during follow-up (mean ± SD, 7.1 ± 3.3 years). Owing to concurrent renal or hepatic comorbidities (two with advanced hepatocellular carcinomas and one with renal urothelial carcinoma with lung metastasis), three remaining AA-group patients refused surgery. They underwent conservative treatment and died within 14 days. Although patients in the CP-group demonstrated high frequencies of comorbidities and progression of PC with increments of mesenteric venous calcifications, colonic wall thickening and involved colonic segments, these patients were efficaciously managed with conservative treatments in long-term follow-up (7.9 ± 4.3 years). Among 14 patients in the CP-group, only two patients underwent right hemicolectomy 4 and 6 years after initial diagnosis owing to obstructive fibrotic stenosis of the ileocecal valve in one patient and pseudo-obstruction of the fibrotic ascending colon with poor motility in another patient (Fig. 1). However, 4 out of 14 patients expired after 3–5 years of follow-up ascribed to other diseases (one with concurrent hepatocellular and renal cell carcinomas, one with bladder cancer and distant metastasis, one with myocardial infarction, and one died in a traffic accident). The clinical status and CT findings of the patients in the CS-group remained unchanged for years (8.2 ± 3.9 years).

## Discussion

Colonic ischemia is caused by markedly reduced blood supply leading to cellular metabolic dysfunction, colonic inflammation, ulceration or eventually necrosis [14, 15]. Venous colonic ischemia is uncommon [15, 16]. PC is a rare form of nonthrombotic venous colonic ischemia almost exclusively seen in individuals of Asian ancestry [1–13]. In contrast to prior studies with male preponderance [7, 8, 11], female predominance (76%) was noted among our patients. Various tools have been utilized for evaluating PC [5–12]. Plain radiographs showed calcified mesenteric veins in 67% of patients in the present study. Thumb-printing of the right colon in enemas and tortuous marginal arteries and vasa recta with occluded mesenteric veins in angiography were found in some of our cases. However, such radiographic studies are not sufficient for PC for the colonic wall, and pericolic and peritoneal status cannot be easily assessed. Colonoscopic delineation of edematous mucosa with dark-purple discoloration is suggestive of PC but presence of ulcerations might mimic ulcerative colitis, and biopsy-induced bleeding could be problematic [8, 11, 12]. Moreover, colonoscopy seems inappropriate for the assessment of PC patients who present with acute abdomen.

To our knowledge, the present study is the first report with long-term follow-up of the clinical findings and temporal CT changes of PC. As seen in our patients, PC could manifest a great variety of symptoms ranging from severe abdominal pain to asymptomatic and thus to obtain a diagnosis solely based on clinical findings seemed impossible in clinical practice. In addition to noninvasive diagnosis of PC by demonstration of characteristic curvilinear mesenteric venous calcifications [5–11], CT could offer colonic wall, pericolic and peritoneal details which were crucial for treatment decision, particularly for those presented with acute abdomen [15, 16]. Our result showed that the AA-group exhibited a significantly thicker colonic wall (mean ± SD, 10.5 ± 3.4 mm), more involved segments and pericolic inflammation than the CP-group and CS-group on initial CT. Such CT findings denoted severe active inflammation of the right colon affirming the decision of early surgery in 7 out of 10 patients, resulting in excellent prognosis. Unfortunately, the remaining 3 patients refused surgery owing to advanced hepatic or renal malignancies died within 14 days and the presence of concurrent PC with detrimental influence in these patients was probable. Of note, long-term CT follow-up in 4 out of 7 surgical patients showed no new mesenteric venous calcifications. In contrast to our results, Lin et al. claimed that venous calcification scores were significantly higher in surgical PC patients, and colonic wall thickening and pericolic fat stranding did not help to identify the need for surgery. However, in Lin’s report, the total calcification score was defined as the summation of the highest calcification scores of 5 major tributaries of mesenteric veins, while the colonic wall was assessed with a categorical analysis with only 3 out 25 of the patients underwent surgery [10]. The present study emphasized the use of summation of the total score of all segments of each calcified mesenteric venous branch and exact measurements of the colonic wall thickness of each edematous segment for ordinal analyses. Nevertheless, even our results yielded a *p* value of 0.046 on initial multiple comparisons of the total calcification scores, subsequent group-to-group comparisons revealed no significant differences between the 3 groups on initial CT.

Patients in the CP-group showed significant increases in mesenteric venous calcifications on long-term follow-up (mean 7.9 years). Yen et al. advocated that the severity of mesenteric venous calcification was associated with the number of episodes of active disease in PC patients [9]. Concurred with Ding et al. [11], we also encouraged the use of CTA for assessing PC by virtue of favorable MIP display or multiplanar reconstruction of venous calcifications making assessment easier and quicker. Our results also verified that PC in CP-patients harbored significant increment of colon wall thickness and more colonic segments involved with distal extension to the left colon during follow-up. However, conservative treatments with a wait-and-watch strategy seemed appropriate, with only two patients eventually necessitating surgery 4–6 years after initial diagnosis owing to fibrotic stenosis of the ileocecal valve and pseudo-obstruction of the fibrotic ascending colon. One patient of PC with involvement of the entire colon has been documented [17], but none of our patients had rectal involvement. In our experiences, CTA assessment for CP-PC patients with acute exacerbation of abdominal symptoms seems mandatary. Of note, 17% of our PC patients showed a benign natural history and remained unchanged for years (mean 8.2yrs). Therefore, for CP- or CS-patients, short-term CT follow-up may not be necessary but long-interval (3–4 years) follow-up may be helpful for monitoring disease progression.

The pathogenesis of PC is controversial. Recently, PC associated with long-term oral intake of geniposide has been reported [12, 18, 19]. Since undigested materials and certain toxic biochemical agents such as geniposide remain static longer in the right colon where the absorption takes place, this may explain the characteristic right colon predominance of PC [18, 19]. However, one unusual case of PC with exclusive involvement of the left colon has been described [20]. Only six of our patients had taken various herbal agents, and no apparent association between herbal medicine and PC could be verified. Diabetes, liver cirrhosis, portal hypertension, hypercoagulopathy, chronic renal disease and immunological abnormalities have also been described to be plausible etiologies of PC [2, 5–12, 21]. Our results revealed that CP-patients demonstrated high frequencies (64–71%) of chronic renal failure, urotract malignancies and liver cirrhosis, supporting the postulation that concurrent renal and hepatic failure could carry potential risk for a chronic-progressive course of PC.

Our study had some limitations. First, since PC is rare, our study had a limited number of patients. Second, the study sample was heterogeneous for various CT facilities were used with evolutional technical differences over long recruitment and follow-up periods. Furthermore, only non-contrast CT were available in some cases. However, no matter single- or multi-detector, non-contrast or contrast-enhanced CT were applied, the assessment of calcifications, colon wall thickening and pericolic inflammation could be undoubtedly achieved in abdominal CT with good image quality. Third, this study was retrospective with potential bias, and some asymptomatic patients with early changes, atypical colonoscopy or imaging features may not be identified. Fourth, it was hard to ascertain why some PC patients experienced acute abdomen while some patients remained quiescent for years and a large-scale study is needed for clarification of this issue.

## Conclusions

Early surgery offered excellent prognosis in PC-related acute abdomen denoted by marked right colonic wall thickening and pericolic inflammation on CT. Conservative treatments with a wait-and-watch strategy were appropriate for CP- and CS-PC, albeit CP-PC harbored frequent comorbidities and significant increases in mesenteric venous calcifications, colonic wall thickening and affected colonic segments in long-term CT follow-up.

## Data Availability

The datasets used and/or analyzed during the current study are available from the corresponding author upon reasonable request.

## Abbreviations

AA: Acute abdomen
CP: Chronic progression
CS: Chronic stable
CT: Computed tomography
CTA: Computed tomographic angiography
MIP: Maximum intensity projection
PC: Phlebosclerotic colitis

## References

[1] Koyama N, Koyama H, Hanagima T, Matsubara N, Fujisaki J, Shimoda T (1991). Chronic ischemic colitis causing stenosis: report of a case [in Japanese]. Stomach Intest.

[2] Yao T, Iwashita A, Hoashi T (2000). Phlebosclerotic colitis: value of radiography in diagnosis-report of three cases. Radiology.

[3] Iwashita A, Yao T, Schlemper RJ (2003). Mesenteric phlebosclerosis: a new disease entity causing ischemic colitis. Dis Colon Rectum.

[4] Kayano H, Nomura E, Hiraiwa S (2016). A case of idiopathic mesenteric phlebosclerosis with progressive intestinal necrosis. Tokai J Exp Clin Med.

[5] Kusanagi M, Matsui O, Kawashima H (2005). Phlebosclerotic colitis: imaging-pathologic correlation. AJR Am J Roentgenol.

[6] Hu P, Deng L (2013). Phlebosclerotic colitis: three cases and literature review. Abdom Imaging.

[7] Fang YL, Hsu HC, Chou YH, Wu CC, Chou YY (2014). Phlebosclerotic colitis: a case report and review of the literature. Exp Ther Med.

[8] Chen W, Zhu H, Chen H (2018). Phlebosclerotic colitis: our clinical experience of 25 patients in China. Medicine (Baltimore).

[9] Yen TS, Liu CA, Chiu NC, Chiou YY, Chou YH, Chang CY (2015). Relationship between severity of venous calcifications and symptoms of phlebosclerotic colitis. World J Gastroenterol.

[10] Lin WC, Chen JH, Westphalen AC (2016). The role of CT in predicting the need for surgery in patients diagnosed with mesenteric phlebosclerosis. Medicine (Baltimore).

[11] Ding J, Zhang W, Wang L, Zhu Z, Wang J, Ma J (2021). Idiopathic mesenteric phlebosclerosis: clinical and CT imaging characteristics. Quant Imaging Med Surg.

[12] Shimizu S, Kobayashi T, Tomioka H, Ohtsu K, Matsui T, Hibi T (2017). Involvement of herbal medicine as a cause of mesenteric phlebosclerosis: results from a large-scale nationwide survey. J Gastroenterol.

[13] Xu J, Jin M, Jiang Z (2020). Clinicopathological features of phlebosclerotic colitis. Pathol Res Pract.

[14] Brandt LJ, Feuerstadt P, Longstreth GF, Boley SJ (2015). American College of Gastroenterology. ACG clinical guideline: epidemiology, risk factors, patterns of presentation, diagnosis, and management of colon ischemia (CI). Am J Gastroenterol.

[15] Duran R, Denys AL, Letovanec I, Meuli RA, Schmidt S (2012). Multidetector CT features of mesenteric vein thrombosis. Radiographics.

[16] Iacobellis F, Narese D, Berritto D (2021). Large bowel ischemia/infarction: how to recognize it and make differential diagnosis? A review. Diagnostics (Basel).

[17] Chen MT, Yu SL, Yang TH (2010). A case of phlebosclerotic colitis with involvement of the entire colon. Chang Gung Med J.

[18] Hiramatsu K, Sakata H, Horita Y (2012). Mesenteric phlebosclerosis associated with long-term oral intake of geniposide, an ingredient of herbal medicine. Aliment Pharmacol Ther.

[19] Wen Y, Chen YW, Meng AH, Zhao M, Fang SH, Ma YQ (2021). Idiopathic mesenteric phlebosclerosis associated with long-term oral intake of geniposide. World J Gastroenterol.

[20] Klein S, Buchner D, Chang DH, Buttner R, Drebber U, Fries JW (2018). Exclusive phlebosclerosis of submucosal veins leading to ischemic necrosis and perforation of the large bowel: first European case. Case Rep Gastroenterol.

[21] Kitamura T, Kubo M, Nakanishi T (1999). Phlebosclerosis of the colon with positive anti-centromere antibody. Intern Med.

